# Breakfast Canyon Discovered in Honeybee Hive Weight Curves

**DOI:** 10.3390/insects9040176

**Published:** 2018-12-01

**Authors:** Niels Holst, William G. Meikle

**Affiliations:** 1 Department of Agroecology, Aarhus University, Forsøgsvej 1, 4200 Slagelse, Denmark; 2 Carl Hayden Bee Research Center, 2000 E Allen Rd, Tucson, AZ 85719, USA; William.Meikle@ARS.USDA.GOV

**Keywords:** monitoring, time series, analysis, segmented linear regression, diurnal, neonicotinoid

## Abstract

Electronic devices to sense, store, and transmit data are undergoing rapid development, offering an ever-expanding toolbox for inventive minds. In apiculture, both researchers and practitioners have welcomed the opportunity to equip beehives with a variety of sensors to monitor hive weight, temperature, forager traffic and more, resulting in huge amounts of accumulated data. The problem remains how to distil biological meaning out of these data. In this paper, we address the analysis of beehive weight monitored at a 15-min resolution over several months. Inspired by an overlooked, classic study on such weight curves we derive algorithms and statistical procedures to allow biological interpretation of the data. Our primary finding was that an early morning dip in the weight curve (‘Breakfast Canyon’) could be extracted from the data to provide information on bee colony performance in terms of foraging effort. We include the data sets used in this study, together with R scripts that will allow other researchers to replicate or refine our method.

## 1. Introduction

**Figure 1:**
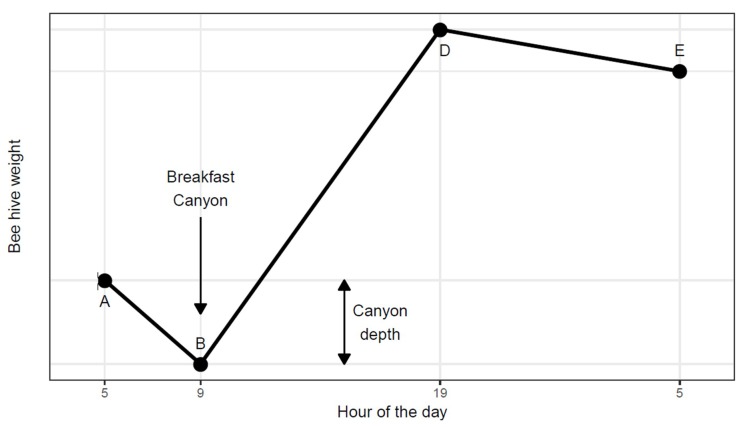
Hambleton’s Graph [1] with Breakfast Canyon added shows the daily pattern of beehive weight during a nectar flow. Abrupt changes in slope occur at A, B, D and E. The canyon has a depth (kg) equal to the weight at A minus the weight at B.

In 1922 Hambleton [1] assigned laborers the task of measuring the weight of two beehives over several months at precise hourly intervals, continuously, day and night. The high-precision scales (±10 g) yielded a unique insight into the daily pattern of hive weight change, resulting from the interplay between the honeybee colony and its surroundings. Hambleton [1] identified a pattern which he summarized in a graph, displaying a series of line segments with clearly defined shifts in slope (Figure 1). After sunrise he found a ‘morning loss’ (from A to B) interpreted as the recruitment of foragers. The morning loss would end abruptly as foragers began returning, thus creating a steady rate of net gain (B to D) due to incoming nectar. He acknowledged that the course of weight change from B to D would vary according to weather and the availability of nectar sources. Between sunset and sunrise (from D to E) he found a constant ‘nocturnal loss’ rate attributed to evaporation and respiration.

The within-day dynamics of beehive weight was not studied further until 70 years later when Buchmann and Thoenes [2] repeated the procedure of Hambleton [1], albeit using electronic scales linked to data loggers. Without recognizing the precedence in Hambleton’s work, they identified the same daily pattern during nectar flows as seen in Figure 1. Later, Meikle et al. [3,4,5] analyzed more extensive data sets of continuously monitored beehive weight but daily variation was reduced to a simple sine wave curve without going into any details of the daily pattern.

The work presented here began as we were intrigued by a regular pattern found in the weight of continuously monitored beehives in Southern Arizona. On many mornings, there would be a distinct dip in weight. This we dubbed ‘Breakfast Canyon’ after a nearby locality, only later realizing that the canyon low point corresponded to the point B of Hambleton (Figure 1).

Continuous weight curves logged at a frequency of 1 h or less is one of several measures used for automatic monitoring of beehive status [6]. If the hive resides constantly on a scale, this monitoring method is nonintrusive. The logged readings of total hive weight are directly linked to the accumulation of honey and pollen stores, which is an important measure of bee colony performance. In addition, the daily amplitude of weight change has been used to infer bee colony size [4,5]. Recently, the within-day pattern of weight changes was analyzed using segmented linear regression [7]. It was concluded that the early morning weight loss was mostly due to foragers leaving.

Here we will address three questions: (i) Is it possible to extract consistently the salient features of Hambleton’s Graph from beehive weight curves? Are the extracted parameters useful for (ii) describing honeybee colony behavior and for (iii) diagnosing neonicotinoid stress? Of these questions, the first two explores the quality and usefulness of the extracted parameters, while the last one forms a hypothesis that can be rigorously tested. 

We used two earlier-published data sets [4] for the analysis from which we estimated three daily parameter values for each beehive: (1) nocturnal loss rate (kg/h), (2) depth of Breakfast Canyon (kg) (Figure 1), and (3) daily weight gain (kg/d). Questions (i) and (ii) were explored graphically while for question (iii) the three parameters were used as response variables, regressed upon the level of neonicotinoid stress brought upon the honeybees in the experiments [4]. The three parameters were successfully extracted by a common R script and provided detailed information on honeybee colony behavior, in general, as well as under neonicotinoid stress. 

## 2. Materials and Methods

### 2.1. Beehive Data Sets

Details of the two experiments that generated the data used in this paper are described elsewhere [4]. Both data sets originate from experiments conducted in the desert climate near Tucson, Arizona, USA to assess the effect of a neonicotinoid insecticide (imidacloprid) supplied regularly in syrup fed to the bees. Hive weights were monitored continuously at 15-min intervals.

The first data set (2014) encompassed 12 beehives for the period 20 April to 17 November 2014. Treatments began 15 July with control (0 ppb), low (5 ppb) and high (100 ppb) doses of insecticide fed in 1:1 sugar syrup with four beehives in each treatment group. The second data set (2015) encompassed 16 beehives for the period 2 May to 14 August 2015. Treatments began 10 July with control (0 ppb), low (5 ppb), medium (20 ppb) and high (100 ppb) doses of insecticide, again with four beehives in each treatment group. In the 2015 data set, data from one medium treatment hive were discarded due to wild animal disruption. The experiment as a whole was cut short due to robbery by a black bear.

### 2.2. Data Preparation

Data from dates when the hives had been manipulated (e.g., by feeding or hive inspection) were discarded from the analysis. Likewise, dates with unexplained, sudden gains in weight were discarded; presumably these were days with heavy rainfall. A few abrupt changes in weight remained, usually due to the checking of equipment; data from these hive × date instances were also discarded. The logged weight was sometimes punctuated by aberrant readings: one or two readings clearly disjoint from readings before and after. These instances were detected algorithmically by the magnitude of weight change, both relatively (kg/h) and absolutely (kg) (see details in the *noise_drop* function found in the *bc-general.R* script in File S2). The disjoint readings were considered missing values. The time stamp of all measurements was transformed from local time to solar time by which solar noon coincides with 12:00; the same algorithm [8] was used to estimate the time of sunrise.

### 2.3. Parameter Estimation

**Figure 2:**
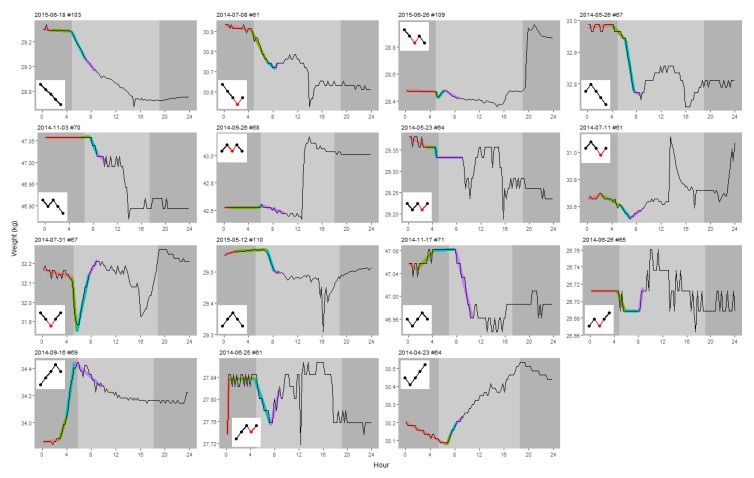
Typical course of the segmented linear regression (SLR) models. For each of the 15 types, the model with the median *r*^2^ value was selected as typical and shown above, identified by date and hive number. The four segments are shown in different colours while the underlying black curve shows the weight during 24 h. Light vs. dark background shows day vs. night. The inset graphical keys refer to Figure 3.

The whole procedure described in the following was automatized in an R script (available in File S2). To describe the daily weight pattern, we used the segmented linear regression (SLR) procedure found in the R package ‘segmented’ [9]. SLR estimates *n* linear regression lines, joined at *n −* 1 breakpoints. An SLR with *n* = 4 segments was carried out for each hive × instance separately on data from midnight to 4 h after sunrise. The ‘segmented’ package uses a stochastic method to search for a solution and thus will not always give the same outcome. Therefore, the analysis was replicated *n* = 30 times. If the analysis failed all *N* times, we tried again with *n* = 60, then *n* = 120 and lastly *n* = 240 times. If no solution was found then the analysis was abandoned for that hive × date instance. Usually more than one solution was found, in which case the solution with the highest *r*^2^ was chosen. Examples of the estimated SLR models are shown in Figure 2. The same procedure, just with *n* = 3 segments, was also carried out but failed to detect many Breakfast Canyon instances obvious to the eye (results not shown).

For each hive × date instance, the SLR model was evaluated for concordance with the Hambleton diagram (Figure 1): An SLR was considered a Breakfast Canyon (BC) SLR, if one of the line segments with a negative slope was followed by one with a positive slope, constituting the two line segments A-B and B-D in Hambleton’s Graph (Figure 1). These could be segments 2-3 or 3-4 but not 1-2, because segment 1 starts at midnight (ruling out that the canyon could commence at midnight). In the 3-4 case, segment 2 could possibly have a negative slope even steeper than segment 3, indicating that segment 2 formed the true onset of the canyon. If so, segments 2-3-4 formed the canyon with the bottom still between segment 3-4.

For every BC SLR model, the nocturnal loss rate (kg/h) was estimated as the negative slope of the longest segment ending at the rim of Breakfast Canyon (point A in Figure 1) or earlier. Canyon depth (kg) was estimated according to Figure 1. For hive × date instances that had no BC SLR, the nocturnal loss rate and canyon depth were considered missing. 

To estimate the weight at midnight, a parabolic curve was fit by linear regression to the data at midnight ±1 h. This resulted in a robust interpolation of weight at 24:00; however, if less than three observations were available in the interval then weight at midnight was considered missing. The weight gain (kg/d) for a particular hive × date instance was found as the increase in the estimated midnight weight from one midnight to the next. 

### 2.4. Statistical Analysis

The quality of the estimated nocturnal loss rates and canyon depths was verified visually by plotting the corresponding extracted line segments. Any line segments with unrealistically steep slopes were identified and discarded from the rest of the analysis. The relations between the three parameters (nocturnal loss rate, canyon depth and weight gain) were explored in three pairwise scatterplots with the aim to hypothesize on plausible explanations for any patterns found. This explorative analysis was followed by a formal statistical procedure to test if the level of neonicotinoid stress had an impact on any of the three parameters. 

We applied a linear mixed model, once for each of the 3 parameters for each year; a total of 6 analyses. The model was formulated in R using the ‘lme’ function:

lme(fixed=Parameter ~ Treatment∗Period, random=~Date|Hive, correlation=corCAR1(form=~Date|Hive))

where Parameter designates one of the three parameters (Breakfast Canyon depth, daily weight gain or nocturnal loss rate), Treatment had 3 levels in 2014 and 4 levels in 2015, and Period had 2 levels designating before or after the onset of the neonicotinoid treatment for that year. The random argument tells that Hive is the subject repeated by Date. The correlation argument specifies the autocorrelation model, which estimated the correlation coefficient (ϕ) between model residuals 1 day apart. Additional details are given in File S1: Section 1.

## 3. Results

After discarding data from dates disturbed by rain and hive manipulations, a total of 3750 hive × date instances remained (2014: 2204, 2015: 1546). The segmented regression procedure successfully estimated 3742 4-segmented SLRs. Thus, it failed in only eight cases. However, for some of the estimated SLRs, the nocturnal loss rate was impossibly large. By experimentation, a threshold of 3.4 s.d. from the average was found to detect these cases. The detected 15 hive × date instances had a loss >97 g/h or a gain >84 g/h, both unlikely to occur at night. They were removed, reducing the data set finally to 3727 instances. Typical examples of the 3727 SLR models are shown in Figure 2.

**Figure 3:**
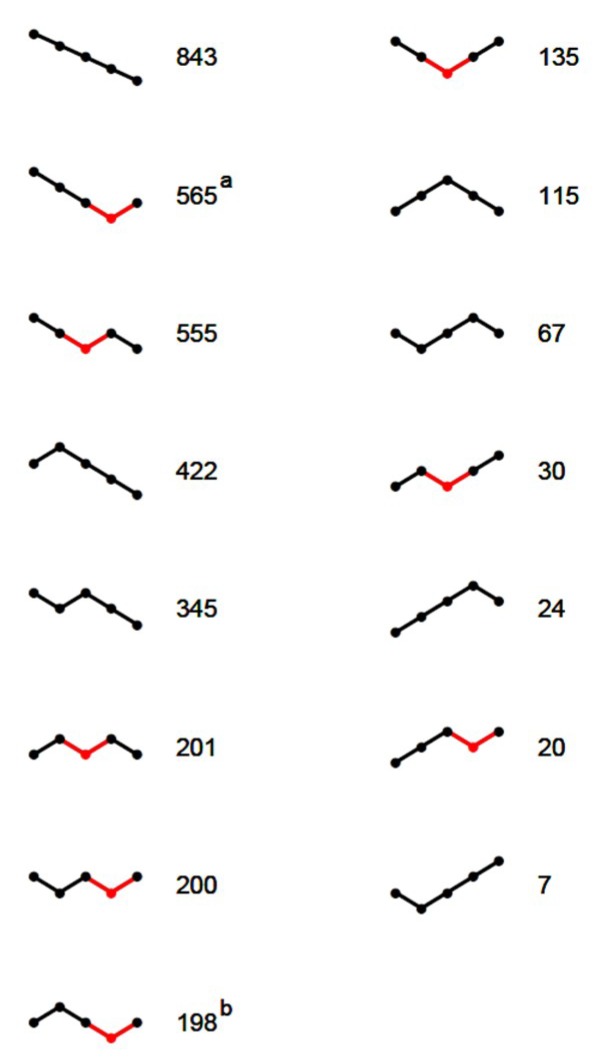
The 15 types of SLR models that best described the weight curves with each type characterized by a sequence of positive and negative slopes. The number found of each type is shown. Breakfast Canyon segments are shown in red—in some cases (a: 174; b: 35) it was extended by one segment to the left (not shown in red).

Depending on the course of the segments (Figure 3), 1904 of the 3727 SLRs (51%) allowed estimation of canyon depth and thereby nocturnal loss rate as well. The remaining 3727 − 1904 = 1823 SLRs, for which a Breakfast Canyon feature could not be identified, were dominated by segments with negative slopes (Figure 3); i.e., losses persisted from the night until 4 h after sunrise when the analysis ended.

**Figure 4:**
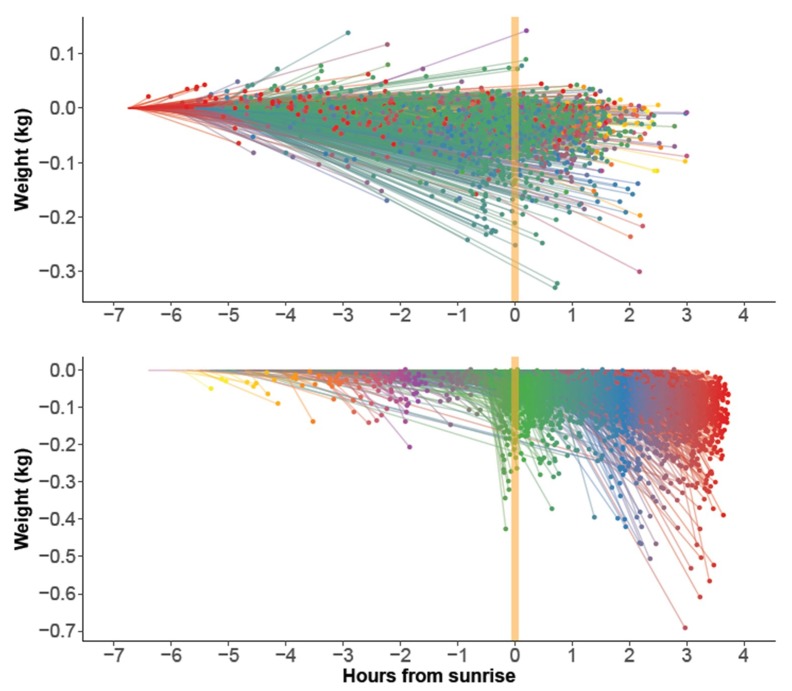
Extracted line segments (*n* = 3727) used to estimate nocturnal loss rate (**top**) and Breakfast Canyon depth (**bottom**). They correspond to lines D-E and A-B, respectively, in Hambleton’s Graph (Figure 1). All segments were translated to begin at zero weight. Line segments and endpoints were color-coded according to the hour of the segment’s beginning to aid the visualization.

The nocturnal loss segments had mostly negative slopes indicating, indeed, a weight loss (Figure 4 top). Weight gains during the night caused by rain had been filtered out before the analysis, so the instances of slight nightly weight gains must have other explanations, maybe light rains or moisture uptake from the air.

The onset of Breakfast Canyon (point A in Figure 1) mostly occurred at ½ hour before sunrise or later (Figure 4 bottom). Canyon bottoms (point B in Figure 1) reached in this interval were also the deepest.

**Figure 5:**
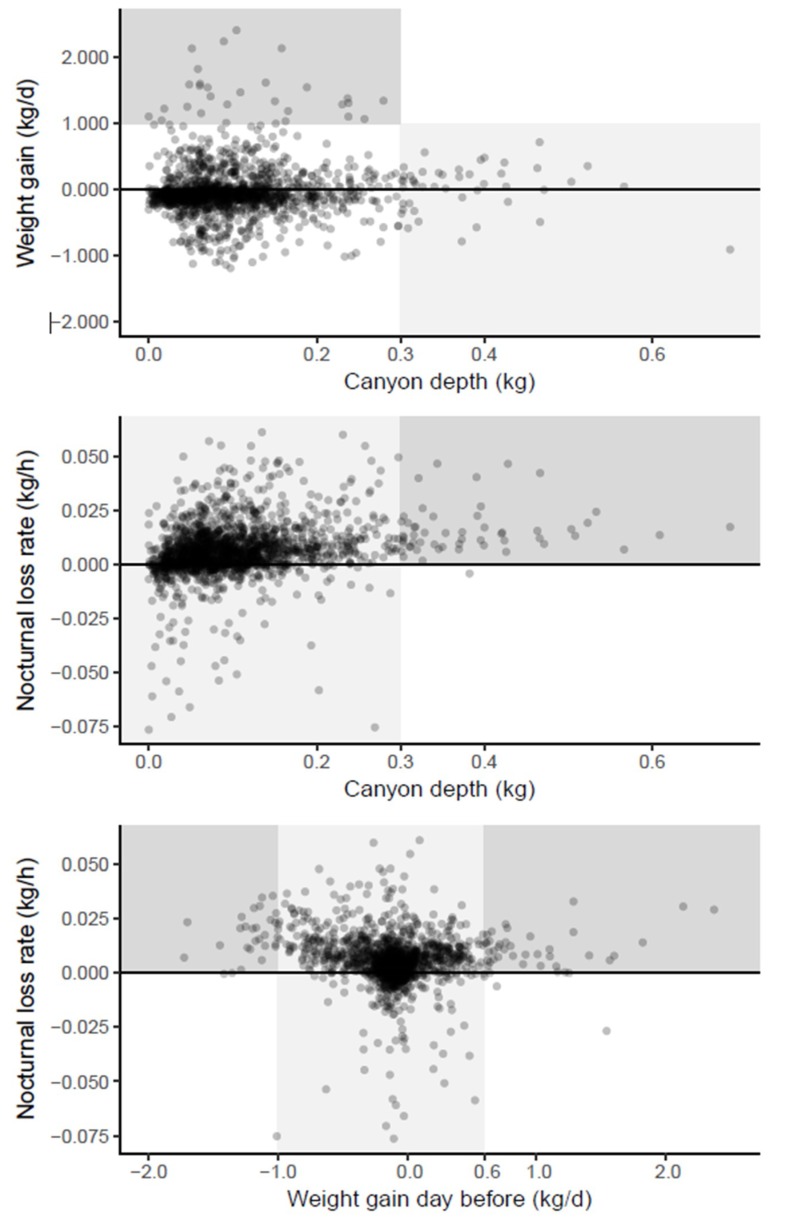
Scatter plots of parameter values estimated for each hive × date instance (*n* = 3732). Weight gain is shown either for the same day (**top**) or for the day before (**bottom**). Light and dark shading shows patterns explained in text.

The scatter plots of the three estimated parameters were inspected for visual patterns which were highlighted by background shading (Figure 5). As these patterns were inspired by the data, they cannot undergo statistical hypothesis testing but only serve to develop plausible biological inferences and hypotheses.

A deep Breakfast Canyon (>0.3 kg) was never followed by a large daily weight gain (>1 kg) (Figure 5 top, light shading). This could be the result of foragers, which on days with scarce resources would spend more time scouting, thus extending and deepening the canyon before they returned with empty crops. In contrast, shallow canyons (depth < 0.3 kg) might be related to rich, nearby nectar sources, resulting in a high weight gain (Figure 5 top, dark shading); or to a low foraging effort, e.g., due to a small number of foragers, resulting in a lower weight gain (Figure 5 top, unshaded points).

A deep Breakfast Canyon (>0.3 kg) only occurred after nights with a distinct weight loss (Figure 5 middle, dark shading). If the weight loss was caused by drying nectar, foragers might have been cued to leave in high numbers anticipating yet another day of high nectar flow.

The nocturnal loss rate was always high when the weight gain during the previous day had been large (>0.6 kg) (Figure 4 bottom, dark shading right). This would be expected since most of the gain would be due to nectar, the main source for evaporation during the night. However, a tremendous loss the day before (more than 1 kg), as well led to high losses at night (Figure 4 bottom, dark shading left). Apparently, these hives suffered a sustained weight loss from the previous day that lasted into the night. For intermediate weight gains during the day (Figure 4 bottom, light shading), rates of gains as well as losses occurred the following night.

**Figure 6:**
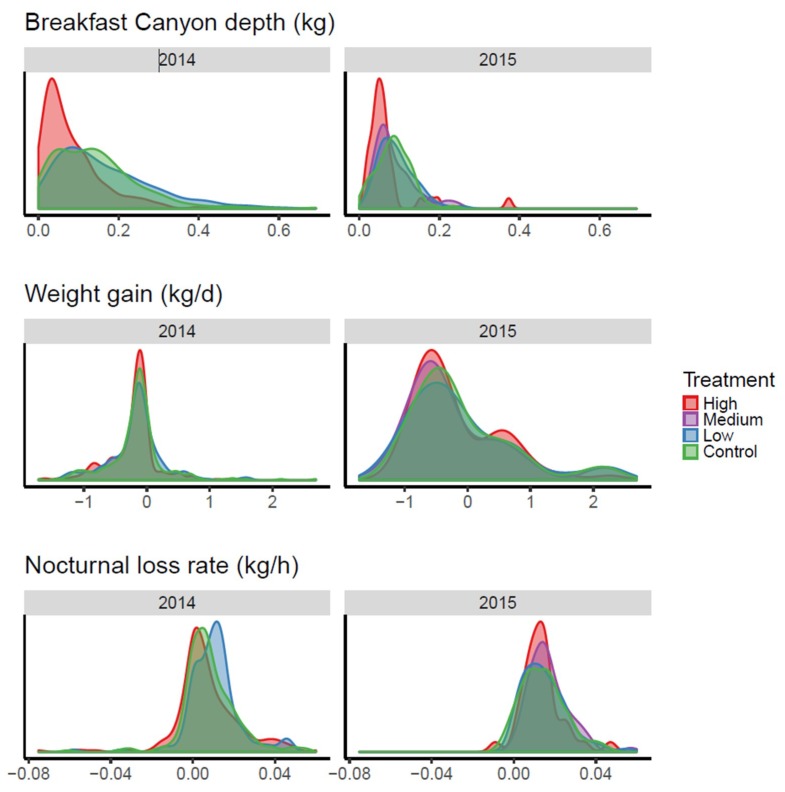
Density plots of daily parameter values estimated in the period after pesticide treatment. The area under each curve equals one. In 2014 there was no medium treatment. Formal statistics presented in File S1: Section 1.

When the three parameters were applied as response variables for the neonicotinoid treatments (Figure 6), the treatment with the highest concentration pushed the distribution towards lower values for Breakfast Canyon depth, making it shallower. The statistical treatment (see File S1: Section 1) supported this result (*p* = 0.003 for both years). The other two parameters seemed not to react to the treatment.

## 4. Discussion

The obvious statistic to extract from beehive weight curves is the daily weight gain e.g., [10,11]. Easily calculated as the weight difference between subsequent midnights, it is directly related to the hoarding of honey and therefore relevant both to the understanding of honeybee ecology and to monitoring of honeybee colony performance. However, through segmented linear regression and subsequent algorithmic analysis, we successfully extracted more detailed information from the daily weight curves, ultimately obtaining consistent estimates of two features of Hambleton’s Graph (Figure 1), namely nocturnal loss rate and Breakfast Canyon (BC) depth. 

The weight changes of a beehive result as a summation of several concurrent processes: nectar inflow and evaporation, fluctuations in honey and pollen stores, base and heating respiration, worker mortality, precipitation, dew, and physical exchanges of humidity between the air and the beehive construction. Pollen stores tend to be short-lived and are not usually stockpiled during periods of ample pollen resources [12,13]. Hence, pollen collection effort is driven by the current demand of the brood [14] and by its availability; the collected pollen is readily consumed. Nectar, on the other hand, is collected both to cover the current energetic demands of the colony and to process and store as honey. This is the basis for ‘nectar flows’, i.e., periods with a steady increase in honey stores. Overall, colonies usually recruit more foragers for nectar collection than for pollen collection [14]. 

Hambleton [1] expected his graph (Figure 1) to apply only to periods of nectar flow. In our study, we identified his graph by a distinctive BC which we found in 51% of the cases. Since we consider nectar flows to have been present less than 20% of the time, Hambleton’s Graph seems applicable also outside nectar flows.

Base respiration will, in general, change only slowly as the colony changes in size and distribution among life stages. In the desert climate of the experiments reported here, respiration must have risen every night to maintain hive temperature in face of the cold outdoors. Total respiration rates of 30−50 g/day have been reported from beehives overwintered at 3–5 °C [15]; we would expect these respiration rates to apply roughly to our hives a well. Water carried in by the bees to cool the hive would not register on the scale, as water is not stored but brought to evaporation immediately. Rain was a rare event but major rainfall events could be detected and were removed from the data set to reduce noise. Thus, disregarding minor showers and physical changes of humidity, major weight changes in these experiments must have been caused by honeybee traffic, together with nectar inflow and its transformation into honey, which involves water evaporation.

The proportion of a bee colony that are foragers varies from 4.1% to 9.6%—larger during nectar flows [16]. The average weight of a worker honeybee has been reported at 115 mg [17] and 128 mg [3]. BC depth varied (Figure 5 top) with a median value of 85 g and a 95% percentile of 249 g. Assuming an average weight of 122 mg for a bee, we get a median BC depth corresponding to 85/0.122 = 697 bees and a 95% percentile of 249/0.122 = 2041 bees. Assuming further that the 95% percentile applies to nectar flow periods, we get an estimate of the total colony size of 2041/0.096 = 21,260 bees. If the median BC depth applies to non-nectar flow periods, which were dominant during the observation periods, we get a total of 697/0.041 = 17,000 bees. The adult bee population in these experiments was typically in the 2–4 kg range [4], corresponding to a colony size of 16,400–32,800 bees (122 mg per bee). Thus, the estimated BC depths are consistent with the hypothesis that BC was caused by bees leaving. 

The mechanisms connecting daily weight gain, nocturnal loss rate and BC depth (Figure 5) were interpreted in terms of honeybee biology. Nocturnal weight gains were not unusual. This points to moisture taken up by the hive structure (made of wood) and by open pollen and nectar cells during the desert night when the relative humidity of the air is rising. In Southern France, empty wooden hives will fluctuate as much as 200 g in weight between night and day [18]; however, in an occupied beehive the fluctuation will likely be less due to the microclimate-regulating habit of the honeybees.

Scout bees will return and recruit foragers through their waggle dance, probably resulting on any given day in a focus on a few high-yielding patches [19]. A potential forager will instead become a scout, the longer time it waits without meeting a recruiting (dancing) bee [20]. In the event that a food patch persists, foragers are likely to remember this and will continue to exploit the same patch without further scouting [21]. These mechanisms will make scouts and foragers out of most of the honeybees leaving during the BC period. If abundant resources are found nearby, the bees will return quickly. This will lead to a shallow BC and a high weight gain on the same day. This mechanism was supported by our data, in which a deep BC never coincided with a high weight gain (Figure 5 top, light shading). 

In the original paper [4] presenting the data reused here, it was found that the ‘high’ neonicotinoid treatment reduced both colony size and average frame weight. We found a reduced BC depth for the high treatment (Figure 6 and statistical analysis in File S1: Section 1), which indicates a lowered foraging capacity of the bee colony. This result supports the original conclusion, that bee colonies were weakened by the high treatment. In conclusion, BC depth might represent a new measure by which to monitor bee colony strength.

We successfully estimated segmented linear regressions in agreement with Hambleton’s Graph (Figure 1) even outside nectar flows. The shape of this graph, however, may vary among climates. Cooler mornings would delay the onset of Breakfast Canyon. A colony placed near a superabundant nectar resource, such as a commercial field of oilseed rape, might shorten the foraging trip as much as to fill up the Breakfast Canyon with returning scouts and foragers, so fast that the canyon would not show; its detection relies on a delay until the first successful bees return. 

In rainy locations, it might be difficult to achieve an undisturbed time series of hive weight. For hives not in some kind of shelter, foraging activity between rain showers would be difficult to filter out from weight fluctuations due to the accumulation of rain on the hive and subsequent drying. For research purposes, the hive and scale should be protected by a rain cover. In addition, hives made of water-inert materials, such as plastic foam, would reduce the noise produced by fluctuations in hive moisture content.

The collection of a variety of sensor data (weight, temperature, humidity, acoustics, digital vision) from honeybee hives has received much attention recently [6]. However, there is a lack of methods to turn the accumulated body of data into biologically meaningful information. The method described here is a step towards establishing a toolbox to turn beehive sensor data into valuable research and monitoring information.

## Supplementary Materials

The following are available online at http://www.mdpi.com/2075-4450/9/4/176/s1, File S1: Application and R script documentation, File S2: R scripts and input files.
