# Supplementary material for: Breakfast Canyon Discovered in Honeybee Hive Weight Curves
Source: Insects. 2018 Dec 1;9(4):176. doi: 10.3390/insects9040176 (PMC6316558; doi:10.3390/insects9040176)
Supplement: Supplementary file 1 [file insects-09-00176-s001.zip › insects-377515-SI/File S1.pdf]

insects

MDPI

# 1. Application

## 1.1. Materials and Methods

We applied a linear mixed model, once for each of the 3 parameters (Breakfast Canyon depth, daily weight gain and nocturnal loss rate) for each year (see Materials and Methods, section 2.4). Thus a total of 6 analyses was carried out with the following model:

$$\begin{gathered}\text{lme(fixed} = \text{Parameter} \sim \text{Treatment} * \text{Period, random} = \sim\text{Date}|\text{Hive},\\ \text{correlation} = \text{corCAR1(form} = \sim\text{Date}|\text{Hive}))\end{gathered} \quad (1)$$

where Parameter designates one of the three parameters. Treatment had 3 levels in 2014 and 4 levels in 2015, and Period had 2 levels designating before or after thxe onset of treatment for that year. The random argument tells that Hive is the subject repeated by Date. The correlation argument specifies the corCAR1 autocorrelation model, which estimated the correlation coefficient (ϕ) between model residuals 1 day apart.

For each analysis, residuals were checked visually in a plot of residuals vs. predicted values (checking variance homogeneity) and in a Q-Q plot (checking normality of residuals). The Parameter (eq. 1) was transformed as necessary, finding appropriate values for *a* and *b* in

$$y = \begin{cases} \tilde{x}^a & \text{for} \quad \tilde{x} \geq 0 \\ -(-\tilde{x})^b & \text{for} \quad \tilde{x} = 0 \end{cases} \quad \text{where} \quad \tilde{x} = \frac{mean(x)}{sd(x)} \quad (2)$$

where the Parameter (represented as x) was standardized before undergoing a power transformation.

In each regression analysis, treatment effects were tested for ($\alpha = 0.05$) by the coefficient for the Treatment × Period interaction term, using the control Treatment and the before-treatment Period as a base line.

**Table S1.** Data set characteristics. Number of observations and autocorrelation (ϕ) at 1-day distance.

| | No. of obs. | | Autocorrelation (ϕ) | |
|---|---|---|---|---|
| Parameter | 2014 | 2015 | 2014 | 2015 |
| Canyon depth | 1336 | 568 | 0.67 | 0.67 |
| Weight gain | 1881 | 1284 | 0.20 | 0.08 |
| Nocturnal loss rate | 1336 | 568 | 0.31 | 0.54 |

## 1.2. Results

Data transformation (eq. 2) was necessary for all 6 mixed regression analyses to obtain normal residuals (*a* ε [0.4; 0.5] and *b* ε [0.5; 1]). The autocorrelation model yielded high correlation coefficients up to $\varphi = 0.67$ (Table S1) which means that temporal autocorrelation was inherent to the data.

The high insecticide treatment led to a more shallow Breakfast Canyon (in both years) and a decreased nocturnal loss rate (2014 only). Treatment had no effect on weight gain in either year. The 2015 data set was smaller (Table S1) giving less statistical power to detect treatment effects, yet canyon depth stood out as a sensitive parameter.

**Table S2.** Treatment effects on three daily beehive parameters. Fixed effects (on transformed scale) from six linear mixed models. Treatment effects are shown as marginal effects ± s.e. after removing control and before-treatment effects. *p*-values less than 0.05 are highlighted.

| | | 2014 | | 2015 | |
|---|---|---|---|---|---|
| **Parameter** | **Treatment** | **Fixed Effect ± s.e.** | **P** | **Fixed Effect ± s.e.** | **P** |
| Canyon depth | Low | 0.025 ± 0.128 | 0.84 | −0.073 ± 0.137 | 0.60 |
| | Medium | n.a. | n.a. | −0.040 ± 0.142 | 0.78 |
| | High | −0.384 ± 0.128 | **0.003** | −0.441 ± 0.147 | **0.003** |
| Weight gain | Low | −0.131 ± 0.216 | 0.54 | −0.017 ± 0.141 | 0.91 |
| | Medium | n.a. | n.a. | −0.129 ± 0.142 | 0.36 |
| | High | −0.362 ± 0.217 | 0.10 | −0.035 ± 0.140 | 0.80 |
| Nocturnal loss rate | Low | 0.028 ± 0.209 | 0.89 | 0.110 ± 0.178 | 0.54 |
| | Medium | n.a. | n.a. | 0.329 ± 0.186 | 0.08 |
| | High | −0.523 ± 0.207 | **0.01** | −0.205 ± 0.194 | 0.29 |

n.a.: Medium treatment not available in 2014.

## *1.3. Discussion*

Earlier work shows that sub-lethal concentrations (20 ppb) of neonicotinoids in supplemental pollen feed make honeybee foragers less willing to leave the hive, as judged by the rate of returning pollen foragers recorded 9 to 11 in the morning [1]. Moreover, neonicotinoids increase the mortality of honeybee scouts and foragers as fewer of them return upon leaving the hive [2]. The first neonicotinoid effect would immediately make BC shallower as fewer bees would be leaving. The second effect would make BC shallower too but in the longer run due to the decimation of scouts. Daily weight gain would decrease under neonicotinoid stress too, both due to the reduced foraging effort of the forager population present and due to a reduction of the forager population itself. The decreased nectar collection and reduced colony size would both lead to a reduced nocturnal loss rate by reducing evaporation and respiration, respectively. Neonicotinoid stress would thus be indicated by a reduction in all three parameters (BC depth, nocturnal loss rate, daily weight gain) estimated from the weight curve.

We found BC depth to be the most sensitive indicator of stress caused by neonicotinoid contamination (Table 2). The 2015 experiment was cut short due to unforeseen events but even so the neonicotinoid effect on BC depth was statistically significant. This indicates that reduced BC depth could be an early warning signal of neonicotinoid contamination, maybe even of reduced colony performance in general. BC became shallower in the high treatment in both years, indicating a reduced foraging effort per capita, or a reduced number of foragers, or both. In contrast daily weight gain was not affected by the treatment (Table 2). However, the nocturnal loss rate declined in the high treatment (Table 2) as could be expected from the mechanisms laid out above. The effect was only evident in 2014 not in 2015, maybe due to the lower number of observations (Table 1).

## 2. R Scripts Documentation

The R scripts found in File S2 read the included input files (Table S3) to produce the output files (Table S4). The R scripts can be executed all together by running the "0-all-steps.R" script as described in the "readme.txt" file also included in File S2.

**Table S3.** Input files included. Found in File S2.

| Input File Name | Content |
|---|---|
| hive-weight-az-2014.txt | Weight (kg) of 12 hives logged 18 Apr 2014–18 Nov 2014 |
| hive-weight-az-2015.txt | Weight (kg) of 16 hives logged 30 Apr 2015–2 Sep 2015 |
| interventions.txt | Calendar of hive interventions noted in 2014–2015 |
| segment-lines.txt | (*x*,*y*) coordinates used to generate line segments for Figure 2 |
| treatments-az-2014.txt | Key to hive insecticide treatments in 2014 |
| treatments-az-2015.txt | Key to hive insecticide treatments in 2015 |
| weather.txt | Hourly weather records for the location of the hives, 2014–2015 |

**Table S4.** Output files generated. Data frames, lists, text files and figures generated by the "0-all-steps.R" script included in File S2.

| Output file name | Content |
|---|---|
| Data frames | |
| best_slr.Rdata | (*x*,*y*) coordinates and $r^2$ for all segmented line regressions |
| canyons.Rdata | Characteristics of all segmented line regressions in which a Breakfast Canyon was detected |
| lme_design.Rdata | Transformations applied in the six *lme* regressions (*cf.* eqs. 1,2) |
| W.Rdata | All weight data on solar time scale. Columns *WeightDetrended* (weight detrended within day) and *WeightCurve* (daily fit of *WeightDetrended* to a sine-cosine wave) are not used in this study |
| WD.Rdata | Daily summary of weight data. *WeightMidnight* and *WeightMidnight2* are the weights estimated at the beginning and the end of the day, respectively. *Amplitude* and *R2* refer to the fit of *WeightCurve*; they are not used in this study |
| WD_canyons.Rdata | Merged canyons and WD data frames. *CanyonR2* refer to the $r^2$ of the segmented linear regression |
| weather.Rdata | Hourly weather records on solar time scale |
| Lists | |
| lme_models.Rdata | Outcome of the six *lme* regressions (*cf.* eq. 1) saved as *lme* objects |
| slr_models.Rdata | Outcome of the segmented line regressions saved as *segmented* objects together with identifiers for hive and date |
| Text files | |
| results.txt | Descriptive statistics referred to in *Results* section |
| table1.txt | Table 1 (unformatted) |
| table2.txt | Table 2 (unformatted) |
| Figures | |
| keys/*.tif | Graphical keys for the different types of segmented linear regressions |
| fig1.pdf | Figure 1 (print-ready) |
| fig2_0.tif | Fig. 2 (to be manually furnished with the graphical keys above) |
| fig3.pdf | Figure. 3 (print-ready) |
| fig4.pdf | Figure. 4 (print-ready) |
| fig5.pdf | Figure. 5 (print-ready) |
| fig6_0.pdf | Figure (redundant legends to be manually erased) |

## References

1. Dively, G.P.; Embrey, M.S.; Kamel, A.; Hawthorne, D.J.; Pettis, J.S. Assessment of chronic sublethal effects of imidacloprid on honey bee colony health. *PLOS ONE* **2015**, *10*, e0118748, doi:10.1371/journal.pone.0118748.
2. Henry, M.; Béguin, M.; Requier, F.; Rollin, O.; Odoux, J.-F.; Aupinel, P.; Aptel, J.; Tchamitchian, S.; Decourtye, A. A common pesticide decreases foraging success and survival in honey bees. *Science* **2012**, *336*, 348–350, doi:10.1126/science.1215039.

© 2018 by the authors. Submitted for possible open access publication under the terms and conditions of the Creative Commons Attribution (CC BY) license (http://creativecommons.org/licenses/by/4.0/).
